# Differential glycosylation of envelope gp120 is associated with differential recognition of HIV-1 by virus-specific antibodies and cell infection

**DOI:** 10.1186/1742-6405-11-23

**Published:** 2014-08-01

**Authors:** Milan Raska, Lydie Czernekova, Zina Moldoveanu, Katerina Zachova, Matt C Elliott, Zdenek Novak, Stacy Hall, Michael Hoelscher, Leonard Maboko, Rhubell Brown, Phillip D Smith, Jiri Mestecky, Jan Novak

**Affiliations:** 1 Department of Immunology, Palacky University in Olomouc, 77100 Olomouc, Czech Republic; 2 Department of Microbiology, University of Alabama at Birmingham, Birmingham, AL 35294, USA; 3 Department of Infectious Diseases & Tropical Medicine, Clinic of the University of Munich, Munich, Germany; 4 NIMR-Mbeya Medical Research Programme, Mbeya, Tanzania; 5 Department of Medicine, University of Alabama at Birmingham, Birmingham, AL 35294, USA; 6 VA Medical Center, Birmingham, AL 35205, USA; 7 Institute of Microbiology and Immunology, First Faculty of Medicine, Charles University, 12108 Prague, Czech Republic; 8 Department of Surgery, University of Alabama at Birmingham, Birmingham, AL 35294, USA

**Keywords:** gp120 glycosylation, Glycan-specific antibody, Deglycosylation resistance, Neutralization inhibition

## Abstract

**Background:**

HIV-1 entry into host cells is mediated by interactions between the virus envelope glycoprotein (gp120/gp41) and host-cell receptors. *N*-glycans represent approximately 50% of the molecular mass of gp120 and serve as potential antigenic determinants and/or as a shield against immune recognition. We previously reported that *N*-glycosylation of recombinant gp120 varied, depending on the producer cells, and the glycosylation variability affected gp120 recognition by serum antibodies from persons infected with HIV-1 subtype B. However, the impact of gp120 differential glycosylation on recognition by broadly neutralizing monoclonal antibodies or by polyclonal antibodies of individuals infected with other HIV-1 subtypes is unknown.

**Methods:**

Recombinant multimerizing gp120 antigens were expressed in different cells, HEK 293T, T-cell, rhabdomyosarcoma, hepatocellular carcinoma, and Chinese hamster ovary cell lines. Binding of broadly neutralizing monoclonal antibodies and polyclonal antibodies from sera of subtype A/C HIV-1-infected subjects with individual gp120 glycoforms was assessed by ELISA. In addition, immunodetection was performed using Western and dot blot assays. Recombinant gp120 glycoforms were tested for inhibition of infection of reporter cells by SF162 and YU.2 Env-pseudotyped R5 viruses.

**Results:**

We demonstrated, using ELISA, that gp120 glycans sterically adjacent to the V3 loop only moderately contribute to differential recognition of a short apex motif GPGRA and GPGR by monoclonal antibodies F425 B4e8 and 447-52D, respectively. The binding of antibodies recognizing longer peptide motifs overlapping with GPGR epitope (268 D4, 257 D4, 19b) was significantly altered. Recognition of gp120 glycoforms by monoclonal antibodies specific for other than V3-loop epitopes was significantly affected by cell types used for gp120 expression. These epitopes included CD4-binding site (VRC03, VRC01, b12), discontinuous epitope involving V1/V2 loop with the associated glycans (PG9, PG16), and an epitope including V3-base-, N332 oligomannose-, and surrounding glycans-containing epitope (PGT 121). Moreover, the different gp120 glycoforms variably inhibited HIV-1 infection of reporter cells.

**Conclusion:**

Our data support the hypothesis that the glycosylation machinery of different cells shapes gp120 glycosylation and, consequently, impacts envelope recognition by specific antibodies as well as the interaction of HIV-1 gp120 with cellular receptors. These findings underscore the importance of selection of appropriately glycosylated HIV-1 envelope as a vaccine antigen.

## Background

The HIV-1 envelope glycoprotein (Env), a trimer of gp120/gp41, is the relevant target for neutralizing antibodies [1-9]. Such antibodies may limit disease progression, as shown for elite neutralizers whose antibodies exhibit strong and broadly neutralizing activity in vitro [9-12]. However, efforts to generate Env-based vaccines have had limited success [13-16] due to the high variability of the *env* gene. For the gp120 subunit, *N*-glycans contribute approximately 50% of the total molecular mass [17]. The viral genome determines the potential attachment sites of the *N*-glycans (specific amino-acid motifs N-X-S/T), whereas the biosynthetic machinery of the host cells producing the virus profoundly determines the composition of the Env glycans [18-20].

We previously reported that glycosylation of gp120 was affected by the cell type and metabolic activity of the producer cells, resulting in distinct gp120 *N*-glycan content and heterogeneity [21]. Notably, gp120 produced in T cells contains mostly high-mannose and hybrid *N*-glycans with fewer complex *N*-glycans compared with gp120 produced in other cell types, such as hepatocytes, which contains a higher proportion of complex *N*-glycans in addition to high-mannose *N*-glycans. The recombinant gp120 produced in T cells, B cells, and HEK 293T cells has extensive heterogeneity of complex *N*-glycans [21]. Moreover, cell-type specific *N*-glycosylation of gp120 affects the binding by antibodies from sera of subjects infected with subtype B HIV-1 [21], results that extend previous work on cell-specific HIV-1 Env glycosylation [17,18,22-28].

HIV-1 attachment to and entry into a host cell requires interaction between viral Env and the host cell CD4 receptor and CCR5 or CXCR4 co-receptor [20,29,30]. Env glycans influence these interactions [31-35] and, thus, impact HIV-1 infectivity [36,37]. In this regard, the vaccine trial RV144 identified a potentially protective epitope of HIV-1 Env in the gp120 V1/2 loop [38,39] and V1/2 loop glycan (N160) that contributes to formation of a quarternary-structure epitope recognized by new class of broadly neutralizing monoclonal antibodies of PG family [10,40]. Although glycans influence protein folding, potentially affecting the conformation of surface-exposed epitopes involved in antibody binding, *N*-glycans also serve as epitopes or part of epitopes for some broadly neutralizing antibodies [40-54]. Conversely, gp120 glycans may serve as a “shield” against neutralizing antibodies [44,55-57]. For example, in late-stage HIV-1 infection, escape variants with *env* gene sequences that encode additional or deleted potential *N*-glycosylation sites (PNGS) promote resistance to neutralizing antibodies [4,31,32,58-69].

In this manuscript, we show that the binding of broadly neutralizing monoclonal antibodies to native, recombinant oligomeric gp120 was determined by the producing cell-specific differential glycosylation. A partial removal of *N*-glycans from gp120 increased the binding of some gp120-specific monoclonal antibodies, as well as polyclonal serum antibodies from persons infected with HIV-1 subtype A/C. Conversely, the binding of several other gp120-specific monoclonal antibodies was reduced. Moreover, the different gp120 glycoforms variably inhibited HIV-1 infection of reporter cells, depending on cell-specific glycosylation. Thus, our data support the hypothesis that the glycosylation machinery of cells that produce HIV-1 Env shapes gp120 glycans and, consequently, impacts antibody reactivity as well as virus infectivity. Therefore, it is important in the design of HIV-1 vaccines to take into account the differential glycosylation of gp120.

## Results

### Glycosylation of gp120 affects recognition by gp120-specific neutralizing and non-neutralizing monoclonal antibodies

To elucidate the role of Env glycans in binding of gp120 by HIV-1-specific antibodies, we evaluated the binding of recombinant gp120 produced in four cell lines (HEK 293T, Jurkat, HepG2, and CHO) representing four differentially glycosylated gp120 variants [21].

**Figure 1:**
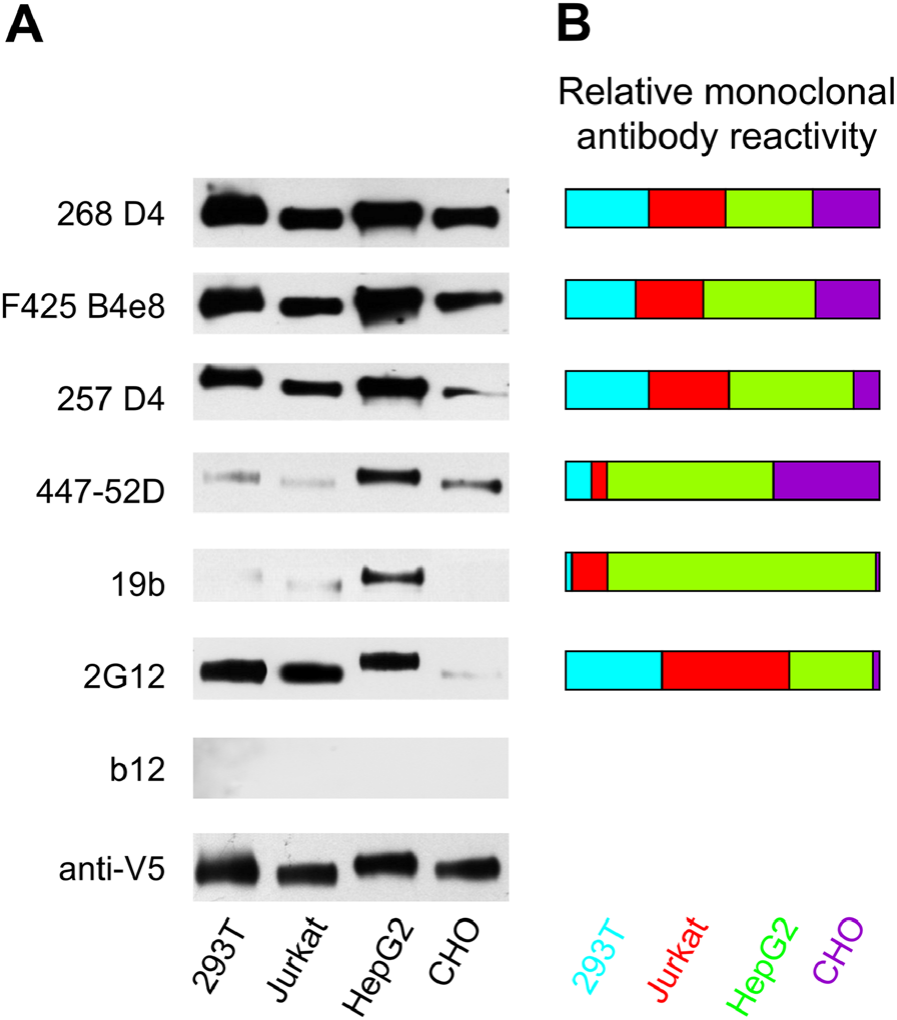
**Reactivities of Env-specific monoclonal antibodies with gp120 produced in different cell lines determined by Western blot. (A)** Antibody binding to gp120 glycoforms produced in HEK 293T, Jurkat, HepG2, and CHO cell lines was analyzed by Western blot after SDS-PAGE separation under reducing conditions. Western blots were developed with monoclonal antibodies 268 D4, F425 B4e8, 257 D4, 447-52D, 19b, 2G12, and b12. Anti-V5-tag antibody was used to assess the amount of loaded gp120. **(B)** Densities of individual gp120 glycoform bands obtained from two Western blots after developing with individual monoclonal antibodies were analyzed by ImageJ 1.41a software. Densitometric values were normalized to loaded amount of individual gp120 glycoforms (V5-tag-positive bands). Mean values from two Western blots were calculated and expressed as relative reactivity of individual monoclonal antibodies with each gp120 glycoform.

Western blot detection after SDS-PAGE separation of denatured and reduced gp120 preparations was performed using two broadly neutralizing (2G12 and b12) and five variably neutralizing (268 D4, F425 B4e8, 257 D4, 447-52D, 19b) HIV-1-specific monoclonal antibodies (Table 1). As shown in Figure 1A, four of the six antibodies (257 D4, 447-52D, and 19b specific to V3 loop, and 2G12 is specific to Manα1,2Man) exhibited clearly visible differences in binding to four gp120 glycoproteins. These data suggested that differential *N*-glycosylation impacted antibody access to the neighboring peptide-based Env epitopes even in denatured and reduced gp120 glycoproteins. The relative differences in antibody binding to the glycoproteins evaluated by densitometric analysis of two independent Western blots are shown in Figure 1B. Notably, gp120 from CHO cells was only marginally recognized by the four antibodies (257 D4, 447-52D, 19b, 2G12), whereas gp120 from HepG2 was preferentially recognized by all tested monoclonal antibodies. Monoclonal antibody b12, which detects a conformational peptide epitope, does not react with gp120 in SDS-PAGE Western blot, and thus served as a negative control.

**Table 1:** Epitope specification of tested monoclonal antibodies

**Monoclonal antibody**	**Epitope structure**	**Epitope**	**Direct involvement of glycans in epitope formation**	Different reactivity with individual gp120 glycoforms*	Glycan-shielding effect**	Dilution corresponding to linear range in ELISA (μg per ml)*	**References**
268 D4	V3 loop tip **HIGPGR**	linear	**-**	**+**	**+**	0.03	[51,70]
257 D4	V3 loop stem **KRIHI**	linear	**-**	**+**	**+**	0.01	[51,70]
F425 B4e8	V3 loop tip involving **GPGRA,** Ile^309^, and Phe^317^	linear	**-**	**-**	**+**	0.04	[52,71]
447-52D	V3 loop tip **GPGR** plus amino acids at N-terminal segment of the V3	conformational	**-**	**-**	**+**	0.07	[72,73]
19b	V3-loop tip **-I----G--FY-T**	linear	**-**	**+**	**+**	0.01	[74]
2G12	Man α1,2 Man-containing oligo-mannose *N-*glycans on C2, C3, V4, and C4	conformational	**+**	**+**	**+**	0.04	[75,76]
PGT 121	V3 base together with multiple surrounding glycans	conformational	**+**	**+**	**-**	0.15	[77]
PG16	Epitope dependent on several glycosylation sites in the V1, V2, V3 loop mostly high-mannose	conformational	**+**	**+**	**-**	7	[45,78]
PG9	Epitope dependent on several glycosylation sites in the V1, V2, V3 loop mostly high-mannose	conformational	**+**	**+**	**-**	6	[45,78]
b12	CD4 binding site	conformational	**-**	**+**	**-**	0.01	[79,80]
VRC03	CD4 binding site	conformational	**-**	**+**	**-**	7	[81,82]
VRC01	CD4 binding site	conformational	**-**	**+**	**+**	0.15	[81,82]

*This study, conclusions based on the differences in mAb binding to individual gp120 glycoforms; ** this study, conclusion based on the partial *N*-glycan removal effect on mAb binding.

**Figure 2:**
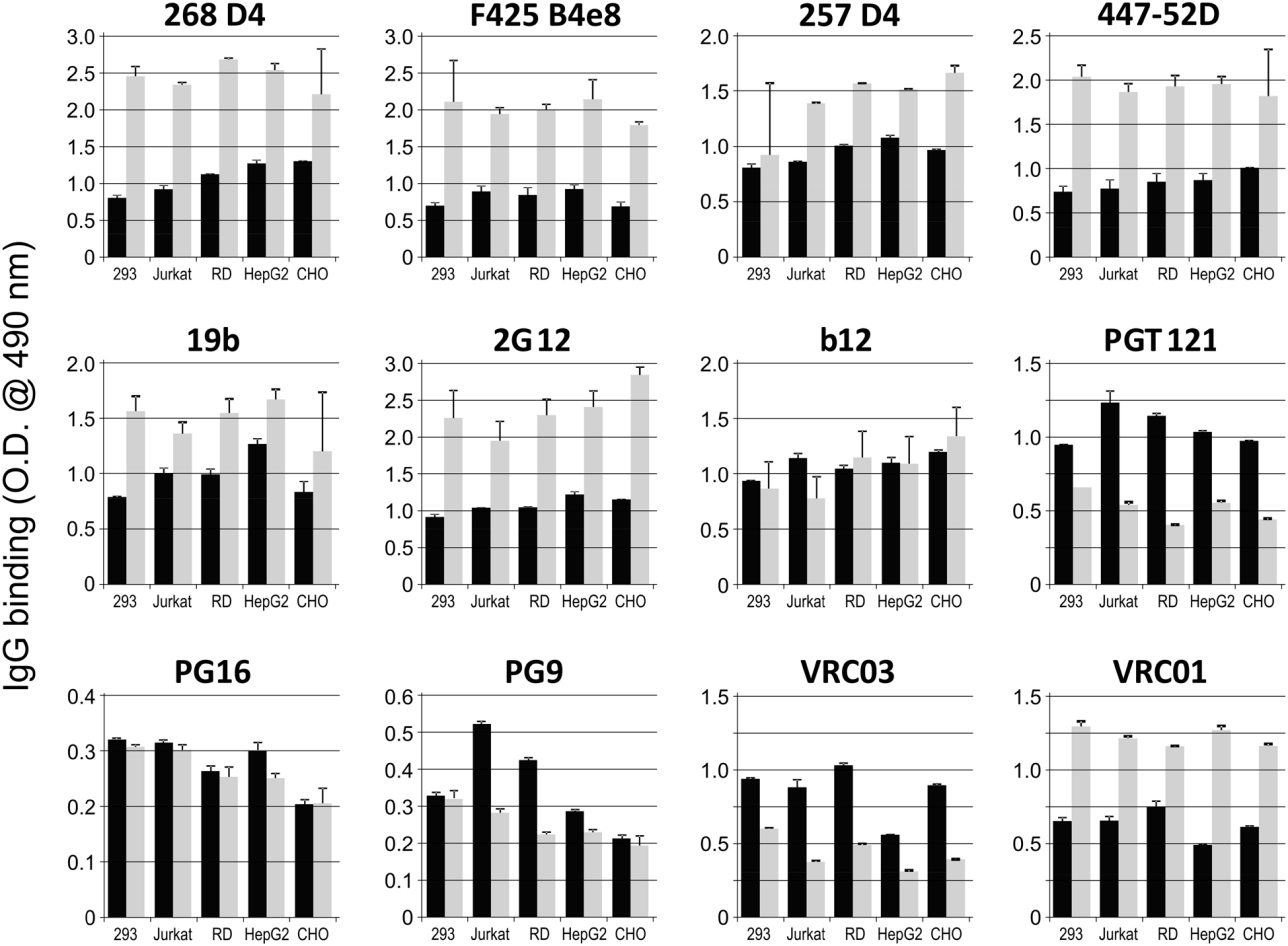
**Comparison of reactivities of selected HIV-1 gp120-specific monoclonal antibodies with native and deglycosylated recombinant HIV-1 gp120 analyzed by ELISA.** ELISA plates were coated with anti-penta-His antibody followed by capture of equal amounts of gp120 produced in HEK 293T (abbreviated as 293), Jurkat, RD, HepG2, or CHO cells (0.05 μg gp120 per well). gp120 preparations were in native form (black columns) or deglycosylated with PNGase F (grey columns). Monoclonal antibodies 268 D4, F425 B4e8, 257 D4, 447-52D, 19b, 2G12, b12, PGT121, PG16, PG9, VRC03, and VRC01 were added and the bound IgG antibodies were detected with HRP-conjugated goat anti-human IgG. Optimal dilutions for each antibody were determined in preliminary experiments using gp120 produced in HEK 293T cells and it is specified in the Table 1. Values correspond to mean absorbance ± SD. Averaged results from three experiments with independently produced recombinant gp120 glycoforms are shown.

Furthermore, we evaluated the binding of HIV-1-specific monoclonal antibodies to native recombinant gp120 produced in HEK 293T, Jurkat, RD, HepG2, and CHO cells using ELISA. Ten of the twelve antibodies (268 D4, 257 D4, 19b, 2G12, b12, PGT121, PG16, PG9, VRC03 and VRC01) demonstrated significant differences in binding to individual gp120 glycoforms (ANOVA, P < 0.02) (Figure 2), confirming the effect of *N*-glycans on the presentation and/or accessibility of the Env epitopes. No significant differences in the binding of the remaining two antibodies (F425 B4e8 and 447-52D; ANOVA, P > 0.17) to the tested gp120 preparations were observed. As the antibodies F425 B4e8 and 447-52D recognize short linear epitope GPGRA and GPGR, respectively, on the apex of gp120 V3 loop [53], the data suggest that those epitopes are not significantly affected by heterogeneity of sterically adjacent glycans. GPGR-containing epitope is recognized also by 268 D4 antibody, but this antibody exhibited significant differences in binding to different gp120 glycoforms. Unlike the F425 B4e8 and 447-52D antibodies, 268 D4 antibody recognizes an epitope extended N-terminally and therefore probably more prone to be influenced by adjacent glycans.

Together, these results suggest that variably glycosylated glycans sterically adjacent to the V3 loop only moderately contribute to differential recognition of a short apex motif of V3 loop (GPGR), but they affect more significantly the binding of antibodies specific for a longer peptide motif that overlaps with GPGR epitope. In contrast to V3 apex-recognizing antibodies, recognition of gp120 glycoforms produced by different cell lines by all other tested monoclonal antibodies specific to epitopes, such as CD4-binding site (VRC03, VRC01, b12), discontinuous epitope involving V1/V2 loop with the associated glycans (PG9, PG16), and V3-base-, N332 oligomannose-, and surrounding glycans-containing epitope (PGT121), was significantly affected (ANOVA, P < 0.01).

The gp120 produced by HepG2 was strongly recognized by five of the twelve monoclonal antibodies (ANOVA, P < 0.01) of which four antibodies are specific to epitopes close to the apex of V3 loop. Notably, gp120 produced by HepG2 cells, which contained relatively low levels of high-mannose *N*-glycans [21], reacted strongly with 2G12, indicating that the discontinuous epitope composed of high-mannose *N*-glycans (Manα1,2Man), as well as folding of the protein, are maintained even in gp120 glycoforms with a high proportion of complex *N*-glycans. The gp120 produced by HEK 293T cells (cells commonly used for production of recombinant gp120 and viruses for HIV-1 studies) exhibited the weakest reactivity with most of the tested monoclonal antibodies, except the CD4-binding-site-specific antibodies (VRC03 and VRC01) and the V1/V2- and V3-loops and associated glycans-specific antibody (PG16) (ANOVA, P < 0.01; Figure 2).

When the binding patterns of the monoclonal antibodies specific to V3 loop apex-containing epitopes determined by Western blot (Figure 1) and ELISA (Figure 2) were compared, the results suggested similar overall dominance in the recognition of gp120 glycoforms from HepG2, although the SDS-denaturation and 2-mercaptoethanol reduction of gp120 during electrophoresis could affect presentation of gp120 epitopes. By comparing the profile for each antibody binding to individual gp120 glycoforms, we found that only 19b antibody, specific for the V3 apex, reacted similarly in both Western blot and ELISA. The gp120 glycoforms from CHO cells were recognized weakly, whereas gp120 glycoforms from HEK 293T cells were well recognized in Western blot although they were among the most weakly recognized gp120 glycoforms in ELISA. Control b12 antibody, which is specific for a multi-peptide conformational epitope [79], did not bind any of the gp120 glycoproteins in the Western blot, due to antigen denaturation, but it reacted strongly with each gp120 in the ELISA. These results suggest that, except of V3 loop apex-specific monoclonal antibodies, the binding of antibodies recognizing epitopes that include CD4-binding site, V1/V2 or V3 base, or specific glycans (such as N160, N332) is dependent on cell-type specific glycosylation of gp120.

### Partial deglycosylation of gp120 affects recognition by some gp120-specific neutralizing and non-neutralizing monoclonal antibodies

**Figure 3:**
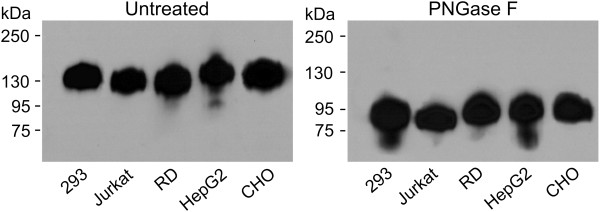
**Mobility shift of gp120 after deglycosylation.** gp120 glycoproteins produced in HEK 293T, Jurkat, RD, HepG2, and CHO cells were deglycosylated by PNGase F under native conditions, separated by SDS-PAGE under reducing conditions, and detected with anti-V5-tag monoclonal antibody. Change in migration of untreated (left panel) *vs.* PNGase F-treated (right panel) gp120 preparations was approximately from 130 kDa to 85 KDa. Results from one of two experiments are shown.

Furthermore, we investigated the contribution of Env *N*-glycans to gp120 recognition by monoclonal antibodies by comparing the antibody binding to the native *vs.* deglycosylated gp120 variants. After deglycosylation of individual gp120 variants using *N*-glycanase (PNGase F) under native conditions, the level of deglycosylation of each gp120 protein was confirmed by mobility-shift assay using SDS-PAGE Western blot (Figure 3). Deglycosylation under optimized conditions (7.5 IUB PNGase F per 250 ng of gp120 protein) substantially reduced the apparent molecular mass of each glycoprotein from 125–135 kDa to 80–90 kDa, indicating that the remaining glycans were resistant to the enzymatic removal under native conditions (Figure 3). Extending the incubation time or increasing PNGase F concentration did not lead to further reduction of the apparent molecular mass of gp120 on SDS-PAGE Western blot (data not shown).

After enzymatic deglycosylation, the binding of all antibodies, except that of b12, changed significantly for a set of five tested gp120 glycoforms (paired *t*-test, P < 0.03). The monoclonal antibody b12 reacted equally well with native and deglycosylated gp120 glycoforms indicating that the contribution of glycans removed under native conditions is negligible for this antibody. Deglycosylation enhanced significantly (paired *t*-test, P < 0.002) the binding reactivity of the V3-loop-specific antibodies (268 D4, F425 B4e8, 257 D4, 447-52D, and 19b) [43,75,83] and CD4-binding-site-specific VRC01 antibody [81]. These data suggest that some glycans partially protect gp120 against antibody binding and that the deglycosylation removes this “glycan shield” (Figures 2A, B). Furthermore, after deglycosylation, the differences in V3-specific monoclonal antibodies reactivities with individual gp120 glycoproteins were not significant. Notably, deglycosylation enhanced more than two-fold ELISA reactivity for 2G12 (paired *t*-test, P < 0.001), a monoclonal antibody specific for clusters of Manα1,2Man-linked glycans, indicating that, in agreement with previously published data, the high-mannose glycans involved in the epitope formation are resistant to PNGase F [84,85]. After deglycosylation, 2G12 recognized each of the five gp120 preparations at significantly different magnitudes (ANOVA, P < 0.007). CHO-produced gp120 antigen was recognized by 2G12 as the best antigen whereas gp120 produced by Jurkat cells was weakly recognized (Figure 2). Conversely, deglycosylation of gp120 under denaturing conditions resulted in loss of 2G12 reactivity as detected by Western blot analysis, as all high-mannose glycans were removed [21]. Unlike the uniform increase in the reactivity of V3-loop-specific monoclonal antibodies after partial deglycosylation of gp120 (increase ranging from 1.5- to 2.5-fold mean ELISA O.D. values; Figure 2), the reactivity of PGT121 (recognizing V3 base, the N332 oligomannose glycan, and surrounding glycans), decreased after gp120 deglycosylation about 0.5-fold compared to the untreated protein (paired *t*-test, P < 0.001), indicating glycan contribution to PGT121 epitope formation. Furthermore, after partial deglycosylation, the variability in PGT121 recognition of individual gp120 glycoforms remained significant (ANOVA, P < 0.001), leading to conclusion that some surrounding glycans involved in epitope formation are still present. Notably, VRC03 and VRC01 antibodies (recognizing CD4-binding site) exhibited differential changes in their reactivities with individual gp120 glycoforms after partial deglycosylation. The reactivity of VRC03 increased about twice (paired *t*-test, P < 0.001), whereas that of VRC01 decreased about 0.5-fold compared to the untreated protein (paired *t*-test, P < 0.001). The differences in the recognition of individual partially deglycosylated gp120 glycoforms remained significant for VRC03 (ANOVA, P < 0.001) and to a lesser extent also for VRC01 (ANOVA, P < 0.021). This finding suggests that the recognition of CD4-binding sites by VRC03 and VRC01 is differentially affected by adjacent glycans and that some of them contribute to CD4-binding-site epitope (VRC03). Moreover, other glycans may mask the access to CD4-binding site (VRC01). Finally, after partial deglycosylation of gp120, the reactivities of two other monoclonal antibodies, PG16 and PG9 (specific for quaternary epitope composed of the gp120 V1/V2 and V3 loops and associated glycans), decreased (paired *t*-test, P < 0.03), although the change was more pronounced for PG9 (Figure 2). The differences in reactivities of PG antibodies remained significant for both PG16 and PG9 (ANOVA, P < 0.004). The most pronounced decrease in PG9 reactivity was observed after deglycosylation of gp120 expressed in T-cell line Jurkat and rhabdomyosarcoma cell line RD. Both cell types are known to produce gp120 with high content of high-mannose glycans [21].

### Glycosylation of gp120 affects the binding of HIV-1-specific serum antibodies to gp120

**Figure 4:**
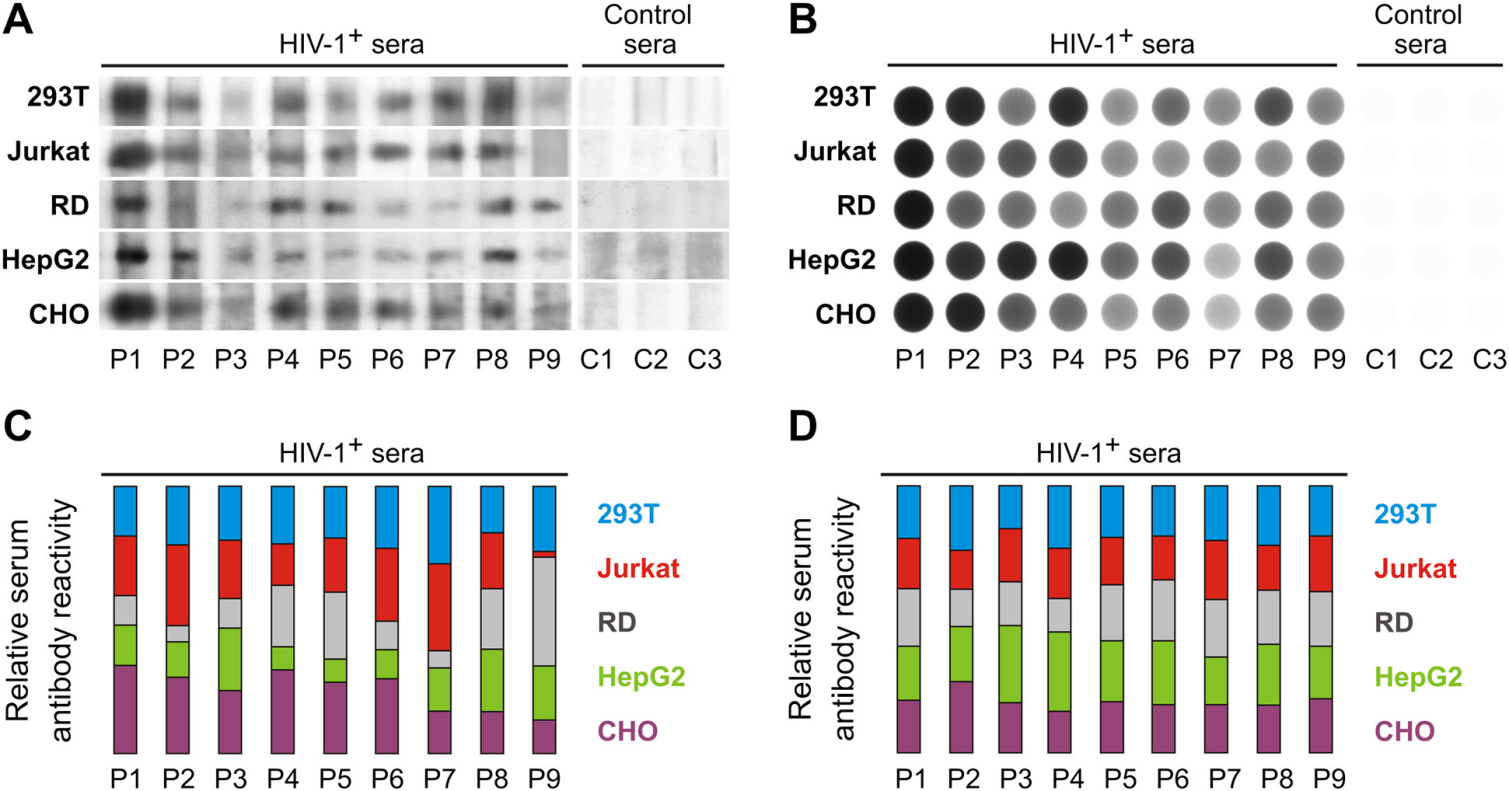
**Reactivities of serum IgG from HIV-1-infected subjects with HIV-1 gp120 produced in different cell lines analyzed by Western blot (A, C) and dot-blot (B, D).** HIV-1 Env gp120 glycoforms produced in HEK 293T, Jurkat, RD, HepG2 and CHO cells were **(A)** separated by SDS-PAGE under reducing conditions and blotted onto a PVDF membrane or **(B)** dot blotted onto a PVDF membrane and both were developed with sera (0.2 μg/ml gp120-specific IgG) from HIV-1-infected subjects (P1-9; HIV-1+ sera) or sera (1:200 dilution) from healthy sero-negative subjects (C1-3; Control sera). Results from one of two experiments are shown. **(C, D)** Densitometric analysis performed with the ImageJ program shows mean reactivity of each serum IgG with individual gp120 variants.

**Figure 5:**
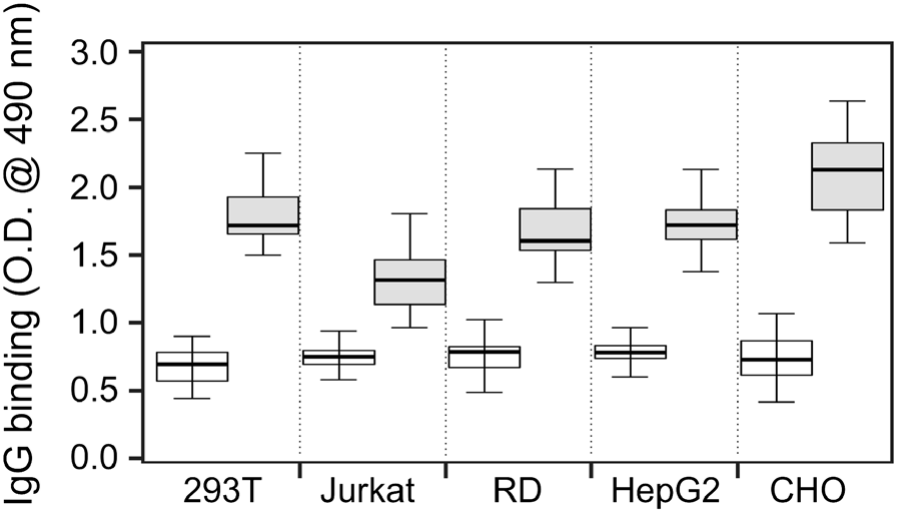
**Reactivities of serum IgG from HIV-1-infected subjects with native and deglycosylated HIV-1 gp120.** ELISA plates were coated with anti-penta-His capture antibody followed by binding of equal amounts of gp120 produced in HEK 293T, Jurkat, RD, HepG2, or CHO cells (0.05 μg per well). Captured gp120 proteins were native (empty boxes) or deglycosylated (gray-filled boxes) using PNGase F under native conditions. Sera from nine HIV-1-infected subjects were added to the ELISA plates at predetermined dilutions (see Methods). Bound IgG antibodies were detected with HRP-conjugated goat anti-human IgG. Data from three independent experiments are shown. Thick line represents median values and boxes the values between 25th and 75th percentiles. The upper and lower whiskers limit 95% of measured values.

Having previously shown that binding of polyclonal antibodies from persons infected with HIV-1 subtype B can differentiate the variably glycosylated gp120 preparations [21], here we investigated the binding of antibodies from sera of individuals infected with HIV-1 subtype A/C to gp120 glycoforms either under denaturing (Western blot) or non-denaturating (dot blot and ELISA) conditions. Serum antibodies from nine subjects infected with clade A/C virus displayed variable recognition of gp120 according to their glycosylation (Figures 4 and 5). Significant differences were observed between SDS-PAGE Western blot (Figure 4A) and dot blot (Figure 4B) in the recognition of specific gp120 glycoforms by antibodies from individual sera. The relative serum antibody reactivities determined densitometrically are presented in Figures 4C and D. Overall differences in recognition of individual gp120 glycoforms by individual sera were higher for Western blot analyses than for dot-blot analyses, indicating that denaturation of gp120 protein could artificially enhance differences in reactivates of serum antibodies, such as detected for sera P2, P4, P6, and P9, although neither reached statistical significance. Furthermore, we used a new ELISA approach that may mimic native conditions, as gp120 oligomers are captured by anti-penta His antibody. The capture antibody serves as a long anchor, thus reducing gp120 contact with hydrophobic surface of ELISA plates. When the recognition of gp120 glycoforms by the same serum antibodies was analyzed by ELISA (Figure 5), the relative binding to individual gp120 differed from the binding detected by Western and dot blots, likely due to gp120 denaturation in the immunoblot assays (Figures 4 and 5). In the Western blot analyses, the best recognized gp120 antigen was that expressed in Jurkat and CHO cell lines, whereas in dot blot analyses the best recognized antigen was expressed in HepG2 cell line. In contrast, ELISA approach did not detect significant differences among reactivities of all nine sera with individual gp120 preparations. These results suggest a strong bias caused by the choice of the method used to detect the binding of HIV-1 Env-specific antibodies to gp120.

Similarly to the increased reactivities observed for surface-exposed-epitope-specific monoclonal antibodies (V3-loop-specific antibodies, Figure 2), partial deglycosylation of gp120 glycoforms significantly enhanced the recognition by serum antibodies (paired *t* test, P < 0.001). Furthermore, the differences in recognition of individual gp120 glycoforms by HIV-1-specific polyclonal antibodies were more pronounced after partial deglycosylation (ANOVA, P < 0.001) compared to untreated gp120 glycoforms (ANOVA, P = 0.154). Notably, gp120 glycoforms generated by Jurkat T cells were recognized less well (ANOVA, P = 0.004) after deglycosylation than the other four glycoforms, although in native conditions all five gp120 glycoforms were recognized similarly (Figure 5). Moreover, antibodies in some of the tested sera exhibited differential reactivity with the gp120 glycans, confirming that gp120 glycoforms produced in specific cell types formed glycan-dependent epitopes.

### Cell-specific glycosylation of gp120 affects HIV-1 infection of indicator cells

**Figure 6:**
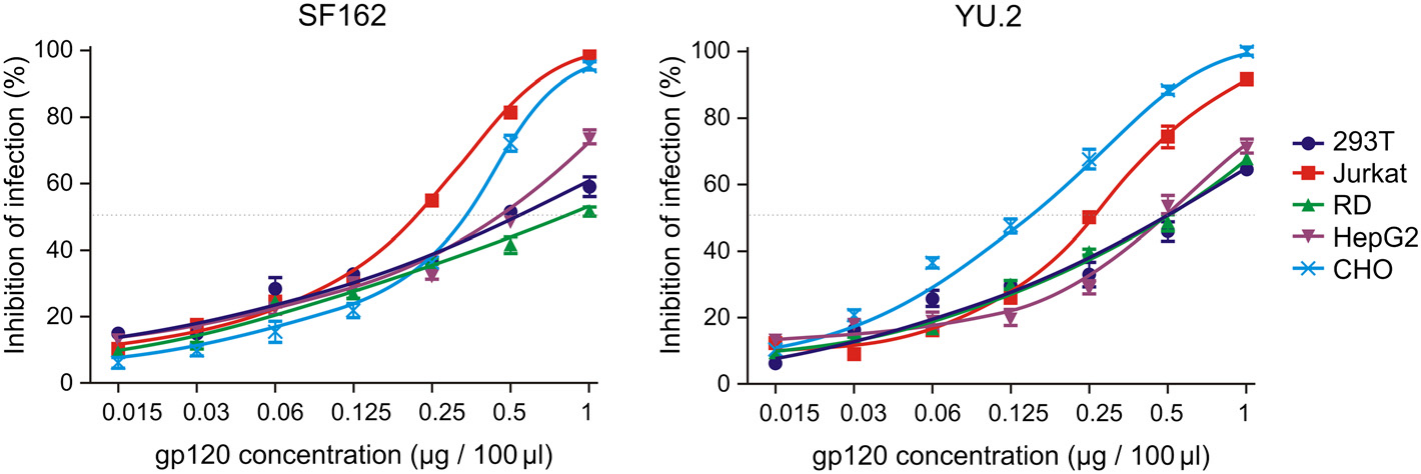
**Inhibition of SF162 and YU.2 HIV-1 infectivity by differentially glycosylated recombinant gp120.** SF162 or YU.2 pseudoviruses (200 TCID50) together with serially diluted gp120 preparations (1 to 0.015 μg/well) were added to TZM-bl reporter cells, and 48 h later luciferase activity was measured in the cell lysates. Results are expressed as % inhibition of the infection of TZM-bl reporter cells, with pseudovirus alone considered 100% infection. Data from three independent experiments are shown.

**Figure 7:**
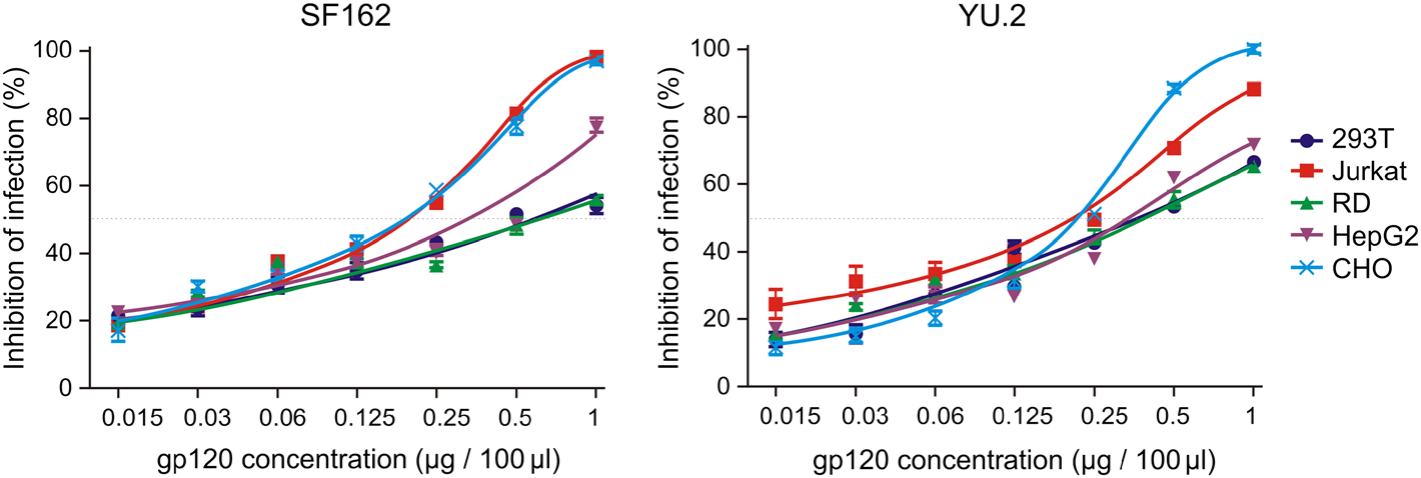
**Inhibition of SF162 and YU.2 HIV-1 infectivity by differentially glycosylated recombinant gp120 preincubated with TZM-bl indicator cells.** SF162 and YU.2 pseudoviruses were added to TZM-bl reporter cells pre-incubated with serially diluted gp120 preparations (1 to 0.015 μg/well). The inhibition of the infectivity was determined and expressed as in Figure 6. Data from three independent experiments are shown.

To determine the effect of differential gp120 glycosylation on HIV-1 infection, we tested the ability of gp120 glycoforms generated in different cell types to block SF162 or YU.2 Env-pseudotyped R5 virus infection of TZM-bl reporter cells. As shown in Figure 6, the IC_50_ for gp120 inhibition of SF162 infection ranged between 0.2 and 0.8 μg/well and for YU.2 infection between 0.1 and 0.5 μg/well. The gp120 produced in Jurkat cells most effectively blocked SF162 virus infection, and gp120 produced in CHO cells most effectively blocked YU.2 infection. In contrast, gp120 produced by HEK 293T and RD muscle cells were the least effective in blocking infection by both viruses. We also pre-incubated the indicator cells with each gp120 preparation followed by addition of either SF169 or YU.2 virus and obtained the same level of inhibition as without pre-incubation (Figure 7). Thus, cell-specific glycosylation influenced the ability of recombinant gp120 to block HIV-1 infection of indicator cells.

## Discussion

In this study, we characterized the cell-specific glycoforms of recombinant trimeric consensus B gp120 with respect to their recognition by monoclonal gp120-specific neutralizing antibodies and polyclonal serum antibodies from persons infected with HIV-1 subtype A/C. For this purpose, we used recombinant gp120 expressed in the N-terminal fusion with a 62-amino acid non-glycosylated fragment of mannan-binding lectin to drive gp120 trimerization and secretion and C-terminal His tag and V5 tag [21,86]. These modifications of gp120 sequence contain only naturally occurring PNGS present in consensus B gp120, and, because they are the same for all preparations, it is unlikely that protein backbone impacted the observed differences in antibody reactivities. The gp120 glycoforms were differentially recognized by the monoclonal as well as polyclonal antibodies. As the gp120 produced in different cell types had the same amino-acid sequence, it implicates that differential cell type-specific glycosylation of gp120 is the solely cause of the variation in antibody binding. Cell-specific differential glycosylation of Env was reported to affect both epitope availability and epitope formation, as *N*-glycans contribute to the proper folding of gp120 [87].

The oligomeric consensus B gp120 that we used in this study is glycosylated, depending on the producer cell type, as we reported earlier [21]. We previously showed that human T cells (Jurkat) produce gp120 with the largest proportion of high-mannose and hybrid *N*-glycans, whereas HepG2 cells secrete gp120 with more complex *N*-glycans and less high-mannose and hybrid oligosaccharides. The greatest variability in glycosylation of gp120 produced by different cell types was related to the relative contribution of complex and hybrid *N*-glycans [21]. Based on the relative abundance of high-mannose oligosaccharides, the oligomeric gp120 expressed in Jurkat and RD resembled, at least partially, gp120 isolated from HIV-1 virions produced by peripheral blood mononuclear cells [56,88].

Here, we extended our previous analyses characterizing differential reactivities of antibodies in sera from HIV-1 clade B-infected individuals [21] to the antibodies in sera from HIV-1 A/C-infected persons and a panel of twelve monoclonal antibodies with five gp120 glycoforms, produced in HEK 293T, Jurkat, RD, HepG2, and CHO cells. Unlike the antibodies in sera from HIV-1 subtype B-infected patients [21], the antibodies in sera from HIV-1 A/C-infected persons reacted similarly with all gp120 glycoforms. We can speculate that the observed similar reactivities of sera from HIV-1 A/C-infected persons with our set of consensus B gp120 glycoforms are due to preferential recognition of highly conserved epitopes on gp120 which are less affected by differential glycosylation than the clade-specific epitopes recognized by sera from HIV-1 clade B-infected persons. Such epitopes likely include also glycopeptides, as several PNGS and the attached *N*-glycans are highly conserved in gp120 of HIV-1 [37,89]. Nevertheless, such glycopeptides are probably not generally recognized by broadly neutralizing antibodies, as the frequency of broadly neutralizing glycopeptide-specific antibodies in HIV-1-infected individuals is quite low [40,44,45,90]. However, when testing the binding of a panel of monoclonal gp120-specific antibodies to gp120 glycoforms, we noted marked differences for some of them. This difference between polyclonal and monoclonal antibodies is probably due to recognition of a broad epitope spectrum by serum antibodies *vs.* single epitope recognized by individual monoclonal antibodies. After partial PNGase F-driven deglycosylation of gp120, the differences in the recognition of individual gp120 glycoforms by serum antibodies increased, probably due to uneven degree of PNGase F-driven deglycosylation and uncovering of several glycan-shielded epitopes. In contrast, the differences in reactivity of monoclonal antibodies with partially deglycosylated gp120 were dependent on localization of the particular epitopes. Specifically, the recognition of gp120 glycoforms by monoclonal antibodies against gp120 V3 loop apex (268 D4, F425 B4e8, 257 D4, 447-52D, 19b) and Manα1, 2Man-linked glycan-specific (2G12) increased markedly. In contrast, partial deglycosylation of gp120 glycoforms differentially impacted the recognition by CD4-binding site-specific antibodies (b12, VRC03, and VRC01): the binding of b12 did not change, VRC03 reactivity decreased, whereas reactivity of VRC01 increased. Recent study using surface plasmon resonance analyses demonstrated that VRC01 binds gp120 with high affinity whereas VRC03 reacts with a 10-fold lower affinity [81]. Thus, VRC01 could act as a partial CD4 agonist in the interaction with gp120, whereas VRC03 does not display this effect [81]. The different VRC03 and VRC01 sensitivity to gp120 deglycosylation observed in this study suggests that VRC03 requires native gp120 glycosylation for effective binding. Due to unchanged proportions in VRC03 reactivities with individual gp120 glycoforms before and after deglycosylation and the observed overall decrease in VRC03 reactivities after deglycosylation, it could be proposed that glycans involved in VRC03 epitope formation are similarly sensitive to PNGase F treatment in all tested gp120 glycoforms. Furthermore, we showed that PG9 and PG16 antibodies, specific to quaternary epitope composed of the gp120 V1/V2 and V3 loops and the associated glycans [40,91], exhibited reduced reactivities with gp120 after partial deglycosylation. Recently, it was reported that changing the glycan profile on the HIV-1 trimer using glycosidase inhibitors or a mutant producer cell line resulted in HIV-1 strain-specific changes in sensitivity to the neutralization activity of PG-family of antibodies [40]. This observation is consistent with disproportional changes in PG9 reactivities with our set of gp120 glycoforms before and after partial glycan removal. The reactivity of another monoclonal antibody tested in our study, PGT121, specific for discontinuous epitope composed of V3 base, the N332 oligomannose glycan and surrounding glycans [47,92], decreased after partial deglycosylation of gp120. This suggests that in contrast to V3 loop which is known to protrude from Env [93], epitopes for monoclonal antibodies which are located in various Env cavities are surrounded by glycans which contribute to epitope formation as well as shielding. This conclusion is supported by the observed changes in reactivities of individual monoclonal antibodies before and after partial deglycosylation of gp120 antigens. Comparison of differences in the reactivities of 2G12 and PGT121 with native and PNGase F-treated gp120 glycoforms suggested that conformational changes in V3 base and/or removal of V1/V2-associated complex glycans could explain the diminished reactivity of PGT121 with PNGase F-treated gp120. Conversely, V3-base glycans or at least some of those glycans involved in binding of 2G12 (N295, N332) are not affected by PNGase F treatment under native condition [47,75,76,83,92].

Our approach could not distinguish between the glycan- *vs.* protein-associated epitopes, as evidenced by the partially retained reactivities of antibodies recognizing glycans on the partially deglycosylated gp120 (PGT121, 2G12, PG16, PG9) [40,47,48,83,91]. However, our tests characterized overall interactions of antibodies with differentially glycosylated gp120 oligomers and discriminated between various glycoforms of gp120 as targets for a panel of well characterized broadly neutralizing monoclonal antibodies. For PGT121, the data suggested that glycans removed by PNGase F under native conditions contributed substantially to PGT121 epitope formation. Moreover, the variability in PGT121 binding to individual gp120 glycoforms after partial deglycosylation suggested that some glycans were still present and involved in epitope formation (Table 1). This interpretation is consistent with our previous finding that complete removal of all glycans requires denaturing conditions [21] as well as with reports of others [76] indicating that some glycans in oligomeric gp140 and gp120/41 Env can be resistant to endoglycosidase cleavage under native conditions.

A partial removal of *N*-glycans reduced differences in monoclonal antibody binding to native gp120, indicating that differential *N*-glycosylation affected formation and/or accessibility of Env-specific epitopes, extending previous observations for monoclonal antibody 19b [49,50]. In contrast, the binding of polyclonal antibodies from sera of subtype A/C HIV-1-infected subjects to native recombinant gp120 glycoproteins was similar for seven of nine sera analyzed by ELISA. However, after partial removal of *N*-glycans binding differences were detected for eight of the nine sera. The most notable increase in reactivity of polyclonal antibodies occurred after deglycosylation of gp120 produced by CHO and HEK 293T (Figure 5), the cells commonly used for production of vaccine antigens for both experimental and clinical trials, including the first partially successful RV144 trial [38,94]; this observation supports the concept that glycans may shield various Env epitopes [4]. The antibody reactivity after gp120 deglycosylation was least pronounced for gp120 produced by T cells, the primary host cell type infected by HIV-1. Together, these findings underscore the importance of the cell type for the production of gp120 as a vaccine antigen for eliciting neutralizing antibodies or binding antibodies for antibody-dependent cell-mediated viral inhibition (ADCVI) [95].

Antibodies specific for glycans are associated mostly with IgG2 and IgA2 subclasses, whereas antibodies specific for proteins are predominantly IgG1 and IgA1 isotypes [96]. Thus, for vaccination purposes, the glycosylation of gp120 antigen should be considered in the strategies to induce isotype-specific immune responses in the systemic and mucosal compartments. In this regard, the results of the recent RV144 trial suggest that IgG antibodies targeting V1/V2 gp120 region may have contributed to protection against HIV-1 infection, whereas high levels of Env-specific IgA antibodies in serum may have mitigated the effects of protective antibodies [39,97]. Studies addressing this point are in progress in our laboratories.

To test the hypothesis that differential gp120 glycosylation affects HIV-1 entry into host cells, and consequently infectivity, we analyzed the capacity of recombinant gp120 glycoforms to inhibit the in vitro infection of reporter cells by two HIV-1 pseudotyped viruses, SF162 and YU.2. The gp120 produced in Jurkat and CHO cells was the most effective at inhibiting infection by both HIV-1 strains. As we reported earlier, the greatest variability in glycosylation of gp120 produced by different cell lines was the relative abundance of complex and high-mannose and hybrid *N*-glycans. Notably, the two gp120 glycoforms from Jurkat and CHO cell lines contained less complex and hybrid glycans compared to high-mannose glycans [21], resembling the glycan patterns of gp120 on virions [56,88]. Potentially a more direct and biologically relevant way to test the effects of cell-type specific glycosylation of HIV-1 gp120 would be to produce HIV-1 pseudotyped viruses in different cell types and then characterize the differences in their infectivity or sensitivity to broadly neutralizing antibodies. Although we attempted this very challenging approach, we have not succeeded to produce sufficient amount of pseudotyped viruses for our experiments, in contrast to the production of recombinant gp120 in stably transfected cells. Furthermore, it would not be possible to unambiguously attribute all potentially observed changes to cell-type specific glycosylation differences because each cell type may be distinctly sensitive to HIV-1 viral regulatory proteins. The main goal of this study was to assess how the differences in glycosylation, according to the producer cell type, affect recognition by HIV-1 gp120-specific neutralizing and serum antibodies and how they affect interaction with HIV receptors on the reporter cells.

In conclusion, although the production of recombinant gp120 is higher in standard cell types used for biotechnology applications (e.g., HEK293), other cell types, such as T cells (Jurkat) produce gp120 antigen with a glycosylation profile resembling that on HIV-1 virions [21]. Jurkat-produced gp120 is well recognized by broadly neutralizing monoclonal antibodies, including the glycan-dependent antibodies PG9 and PGT121, and effectively competes with HIV-1 virions in binding the receptors on reporter cells*.*

## Conclusion

Together our data revealed that: Recognition of gp120 by V3-loop-specific monoclonal antibodies was hindered by differential glycosylation of gp120; The recognition of CD4-binding sites by VRC03 and VRC01 was distinctly affected by neighboring glycans, with some of them contributing to CD4-binding site formation (VRC03) and others masking the access to CD4-binding site (VRC01); The reactivity of PGT121 (recognizing V3 base, the N332 oligomannose glycan, and surrounding glycans) was reduced after partial *N*-glycan removal, indicating that some *N*-glycans contribute substantially to PGT121 epitope formation; Differences in reactivities of polyclonal serum antibodies from subjects infected with HIV-1 subtype A/C with individual gp120 glycoforms were modest, unlike those observed for subjects infected with HIV-1 subtype B. However, the reactivites increased after partial glycan removal for some gp120 glycoforms; The gp120 produced by T cells and CHO cells most effectively blocked infection of reporter cells by HIV-1 virions.

In summary, we show that cell type-specific glycosylation of oligomeric gp120 impacts recognition by serum antibodies from HIV-1-infected subjects and by monoclonal gp120-specific antibodies. Furthermore, cell type-specific glycosylation of gp120 affects its ability to compete with HIV-1 and inhibit infection of TZM-bl reporter cells. This suggests that glycosylation influences the binding of gp120 to cellular receptors/co-receptors and consequently affects the receptor-mediated HIV-1 infection of target cells. Cell type-specific differential glycosylation is characterized by variability in the number and content of complex and high-mannose/hybrid *N*-glycans. Importantly, T cells produced gp120 glycoforms similar to the high content of high-mannose and hybrid glycans found on gp120 of isolated virions [56]. Thus, the glycosylation machinery of cells that produce gp120 shapes its glycosylation and, consequently, impacts antibody reactivity, underscoring the importance of differential gp120 glycosylation in the design of HIV-1 Env vaccines.

## Methods

### Human sera

Serum samples were obtained from subjects infected with HIV-1 subtype A and/or C not receiving anti-retroviral therapy in the Mbeya Region of Southwestern Tanzania (HIV Superinfection Study; HISIS), as detailed in previous publications [98-100]. HIV-1 gp120-specific titers of serum IgG antibodies were measured by ELISA, as previously reported [100].

Monoclonal anti-gp120 antibodies 268 D4 (recognizes epitope HIGPGR on V3 loop), F425 B4e8 (recognizes epitope GPGRA on V3 loop), 257 D4 (recognizes epitope KRIHI on V3 loop), 447-52D (recognizes epitope GPGR on V3 loop), 19b (recognizes apex of V3), 2G12 (recognizes discontinuous epitope composed of clusters of Manα1,2Man-linked glycans on the “silent face” of gp120), b12 (recognizes an epitope overlapping the CD4 binding site), PGT121 (recognizes discontinuous epitope composed of V3 base, the N332 oligomannose glycan and surrounding glycans, including a putative V1/V2 complex biantennary glycan), PG9 and PG16 (recognize an epitope consisting of the gp120 V1/V2 and V3 loops and associated glycans), VRC01 and VRC03 (recognize the CD4-binding site) were obtained from the NIH AIDS Research and Reference Reagent Program, Division of AIDS, NIAID, NIH [40,46-54] (Table 1).

### Recombinant HIV-1 gp120 preparations

Human cell lines, including human embryonic kidney cell line HEK 293T, the Jurkat T-cell line, rhabdomyosarcoma cell line RD, and hepatocellular carcinoma cell line HepG2, and Chinese hamster ovary cells (CHO), were obtained from ATCC. Cells were stably transfected with plasmid encoding a codon-optimized consensus B gp120 DNA (GenBank DQ667594 fragment 88–1485) fused N-terminally with cDNA coding for the first 62 amino acids of human mannan-binding lectin (MBL; GeneBank EU596574 fragment 66–252) and C-terminally with His and V5-tag to express the recombinant gp120 in oligomeric form. These modifications of gp120 sequence did not contain any PNGS, and being the same for all preparations have not likely impacted the results. Structure and glycosylation of all five gp120 variants were described previously [21,86]. Each cell clone was used to isolate at least 200 μg of glycoprotein. The gp120 was purified by affinity nickel-nitrilotriacetic acid (Ni-NTA) agarose under native conditions [21]. The concentration of recombinant glycoproteins was determined by BCA protein assay (Pierce, Rockford, IL) and verified by densitometric analysis of Coomassie blue-stained protein bands after SDS-PAGE (BioRad, Hercules, CA) using ImageJ 1.41a software. The recombinant glycoproteins were aliquoted and stored at −80°C.

### ELISA and glycan removal from gp120

To determine the reactivity of monoclonal antibodies or IgG in the sera of HIV-1-infected subjects, ELISA MaxiSorp plates (Nalge Nunc, International, Rochester, NY, USA) were coated overnight with 100 μl of 1 μg/ml mouse anti-penta-His monoclonal antibody (Qiagen, Valencia, CA, USA). After blocking with SuperBlock (Pierce) supplemented with 0.05% Tween 20 (SB-T), 0.05 μg of native gp120 or partially deglycosylated gp120 were added to each well and incubated overnight at 4°C. To remove *N*-glycans without gp120 denaturation, treatment with endoglycosidase PNGase F was optimized to reach maximal glycan removal under native conditions as determined by mobility shift on Western blot (7.5 IUB PNGase F per 250 ng of gp120 protein in PBS, incubated for 24 h). All assays were performed in duplicate or triplicate, depending on the amount of monoclonal antibodies or sera available. Antibodies were titrated in ELISA using recombinant oligomeric consensus B gp120 produced in HEK 293T cells captured on anti-penta-His monoclonal antibody-coated plates. Assays were performed using three dilutions of monoclonal antibodies or sera corresponding to the linear portion of the titration curve; the middle dilution was used for statistical analyses. Antibodies bound to the gp120 were detected with HRP-labeled goat anti-human IgG (Sigma, St. Louis, MO, USA) followed by the peroxidase substrate O-phenylenediamine-H2O2. The reaction was stopped with 1 M sulphuric acid and the absorbance measured at 490 nm [21,101].

To detect possible differences in the binding of native *vs.* deglycosylated gp120 to the anti-penta His antibody-coated wells on ELISA plate, we compared the binding of anti-V5-tag monoclonal antibody to the gp120 preparations. No differences were detected in the binding of anti-V5-tag.

### Inhibition of HIV-1 infectivity by recombinant gp120

Serial dilutions (1 to 0.015 μg/well) of recombinant gp120 in DMEM medium without fetal calf serum were added to ~60% confluent TZM-bl reporter cells containing reporter cassettes of luciferase and β-galactosidase [4,102-106]. Either immediately or after 1 h pre-incubation at 37°C, an equal volume of YU.2 or SF162 Env-pseudotyped R5 virus (200 median tissue culture infective dose, TCID50; corresponding to 150,000 relative luminescence units, RLU) in medium containing 20% FCS and 30 μg/ml DEAE dextran MW 500,000 (Sigma) was added to each well. The viruses were produced in HEK 293T cells [104]. After 48-h incubation at 37°C with 5% CO_2_, 75 μl of lysis buffer (Promega, Madison, WI, USA) was added to each well, and plates were submitted to three cycles of freeze-thaw. The luciferase activity of the cell lysates (25 μl/well) was measured with a luminometer after the addition of luciferase substrate (Promega). The readings of RLU are directly proportional to the number of infectious virus particles, indicating that the reduction in RLU in wells with gp120 reflects blockade of infection. The results are expressed as % inhibition of HIV-1 infectivity of TZM-bl target cells, with virus alone considered 100%.

### SDS-PAGE/Western blots

The gp120 preparations were separated by SDS-PAGE under reducing conditions and blotted on PVDF membranes. Membranes were blocked with SB-T and developed with anti-gp120 monoclonal antibody or mounted into Mini-PROTEAN II Multiscreen Apparatus (BioRad) and developed with sera from HIV-1-infected or healthy control subjects. The HIV-1-positive sera were diluted to a final concentration of 0.2 μg/ml gp120-specific IgG, previously determined by ELISA [100]; control sera from healthy subjects were diluted 1:200, which means ten time less diluted than average dilution used for HIV-1 positive sera to exclude non-specific binding. The PVDF membranes were incubated overnight with monoclonal antibodies or sera, and then developed with HRP-conjugated goat anti-human IgG antibody (Sigma). The peroxidase-positive bands were detected with SuperSignal West Pico (Pierce) and visualized by exposure on X-ray film (Kodak) or by using a cooled CCD camera (Roche Diagnostic Corp., Indianapolis, IN, USA). The HRP-conjugated anti-V5-tag antibody (Invitrogen, Carlsbad, CA, USA) diluted 1:7,000 in SB-T was used as a positive control.

### Dot-blot analysis

To detect gp120-specific antibodies in sera, 50 ng of recombinant gp120 proteins were applied to each well of a 96-well plate with a PVDF membrane (MultiScreen IP Filer Plate; Millipore, Billerica, MA) and incubated overnight at 4°C. After blocking with SB-T, sera diluted as described for the Western blot analysis were added, and the plates were incubated overnight at 4°C. The bound antibodies were detected as described for the Western blot analysis.

### Densitometric analysis

To compare the binding intensity of anti-gp120 antibodies to differentially glycosylated gp120, the densities of bands obtained by Western blot and of dots detected by dot-blot assay were analyzed by ImageJ 1.41a software. Densitometry was performed using calculated background-corrected integrated density over the area adjusted for the largest band or dot of the analyzed gp120.

### Statistical analysis

Differences between groups and statistical significance was determined by utilizing analysis of variance (ANOVA) and paired *t*-test. All statistical analyses were performed using SPSS v. 21 statistical package (IBM Corp., Armonk, NY, USA).

## Abbreviations

BCA: Bicinchoninic acid; Ni-NTA: Nickel-nitrilotriacetic acid; PNGase F: Peptide N4-(N-acetyl-betaglucosaminyl) asparagine amidase F; PVDF: Polyvinylidene difluoride; SB-T: SuperBlock plus 0.05% Tween 20; SDS-PAGE: Sodium dodecyl sulfate polyacrylamide gel electrophoresis.

## Competing interests

The authors declare that they have no competing interests.

## Authors’ contributions

MR participated in the design of the study, performed the dot blot analyses, participated on the deglycosylation-based ELISA assays and drafting the manuscript, LC participated in the ELISA assays and production of recombinant protein, ZM participated in the inhibition assays and drafting the manuscript, KZ participated in the recombinant gp120 purification, ME participated in the Western blot assays, ZN performed the statistical analysis, SH participated in the protein characterization, MH provided the serum samples, LM was involved in volunteer recruitment and sample collection, RB participated on protein purification, PS participated in drafting the manuscript, JM participated in drafting the manuscript, and JN participated in the study design and drafting the manuscript. All authors read and approved the final manuscript.
